# Reducing Serious Hospital-acquired Pressure Injuries in Pediatric Intensive Care Units through Proactive Interdisciplinary Rounding

**DOI:** 10.1097/pq9.0000000000000943

**Published:** 2026-10-28

**Authors:** Rachel E. Gahagen, Kate Lucey, Kenny Kronforst, Jacqueline M. Knell, Kate Chapman, Monica Mindel, John Sarmiento, Lauren M. Weisert, Casey Vogel, Catherine A. Collins

**Affiliations:** From the * Division of Pediatric Critical Care, Ann and Robert H. Lurie Children’s Hospital, Chicago, Ill.; † Department of Pediatrics, Northwestern University Feinberg School of Medicine, Chicago, Ill.; ‡ Division of Hospital Based Medicine, Ann and Robert H. Lurie Children’s Hospital, Chicago, Ill.; § Division of Neonatology, Ann and Robert H. Lurie Children’s Hospital, Chicago, Ill.; ¶ Center for Quality and Safety, Ann and Robert H. Lurie Children’s Hospital, Chicago, Ill.; ‖ Lefkofsky Family Pediatric Intensive Care Unit, Ann and Robert H. Lurie Children’s Hospital, Chicago, Ill.; ** Division of Rehabilitation, Ann and Robert H. Lurie Children’s Hospital, Chicago, Ill.; †† Division of Cardiology, Ann and Robert H. Lurie Children’s Hospital, Chicago, Ill.

## Abstract

**Introduction:**

Hospital-acquired pressure injuries (HAPIs) contribute to significant morbidity, prolonged hospitalization, and increased healthcare costs in pediatric intensive care units (PICUs). Critically ill children are at increased risk due to immobility, complex comorbidities, and frequent use of medical devices. Despite stable rates of adherence to prevention bundles, an increase in serious HAPIs (sHAPIs)—defined as stage 3, stage 4, or unstageable injuries—was observed at our institution. This quality improvement initiative aimed to reduce the rate of sHAPIs by 10% through the implementation of proactive interdisciplinary rounding.

**Methods:**

Weekly interdisciplinary pressure injury rounds were implemented across the cardiac intensive care unit, neonatal intensive care unit, and PICU at a freestanding 364-bed children’s hospital. Rounding teams included physicians, nurses, certified wound ostomy nurses, clinical quality coordinator nurses, and rehabilitation therapists. High-risk patients were identified using the Braden QD Scale and unit-specific criteria. During rounds, teams discussed prevention strategies and implemented targeted interventions, including device optimization, mobility promotion, nutrition assessment, skin protection measures, and bedside education. Outcome, process, and balancing metrics were tracked.

**Results:**

Within 1 year of implementation, the ICU sHAPI rate was reduced by 39% from 0.564 to 0.343 per 1,000 patient-days. The time between events increased in the PICU and cardiac intensive care unit. More than 1,400 proactive interventions were implemented. Rates of non-sHAPI hospital-acquired conditions decreased from 4.8 to 2.8 events per month.

**Conclusions:**

Proactive interdisciplinary rounding was feasible, reproducible across ICUs, and associated with fewer sHAPIs.

## INTRODUCTION

Pressure injuries are areas of tissue damage that occur as a result of compression of a bony prominence on an external surface for an extended period of time.^1^ Hospital-acquired pressure injuries (HAPIs) result in significant morbidity, dissatisfaction, and prolonged length of stay for hospitalized children.^2–4^ Critically ill pediatric patients are at a particularly high risk due to their multiple comorbidities, relative immobility, altered nutrition and metabolic demands, and device requirements.^5^ As such, HAPI risk is prevalent across intensive care settings.^3,6–8^ This prevalence has significance, as a single pressure injury is estimated to add $26,000–$43,000 to the cost of a child’s hospital stay.^4^

In neonatal intensive care units (NICUs), preterm infants and newborns with complex medical conditions are particularly vulnerable to pressure injuries due to skin immaturity, vascular integrity resulting in significant third spacing, need for medical devices, and immobility.^7^ Likewise, in pediatric intensive care units (PICUs), children may develop pressure injuries due to extended periods of immobilization, fluid overload, poor tissue perfusion, and the presence of medical devices such as mechanical ventilators or extracorporeal membrane oxygenation (ECMO) cannulae.^1,9^ Pediatric cardiac intensive care unit (CICU) patients are also at high risk, with pressure injuries frequently occurring near ECMO cannulation sites, chest tubes, and surgical incision sites following cardiac procedures.^5,10,11^ With advances in medical technology, common causes of HAPIs are medical devices such as oxygen saturation probes, intravenous securement devices, noninvasive positive pressure masks, and endotracheal, gastrostomy, and tracheostomy tubes.^12^

The significance of these injuries is both direct and indirect. Stage 3, stage 4, and unstageable pressure injuries acquired after admission are classified as “Serious Reportable Events” by the National Quality Forum.^13^ Similarly, the Centers for Medicare and Medicaid Services regards stage 3 and stage 4 pressure injuries that develop during hospitalization as “hospital-acquired conditions” (HACs). Consequently, hospitals cannot charge additional fees for these cases, resulting in lost revenue from prolonged hospital stays and the cost of treatment. Although these regulations were initially established for Medicare patients, numerous Medicaid providers and private insurance companies have since adopted similar policies.^14^

Despite ongoing hospital-wide quality improvement (QI) efforts to track and reduce pressure injuries, there was an increase in ICU serious HAPIs (sHAPIs) at our institution in 2022–2023. Our institution has had difficulty sustaining adherence to the Solutions for Patient Safety (SPS)^15^ bundle due to its complexity and challenges related to measuring observed versus charted bundle components. Although pressure injury bundles can reduce HAPIs, the certainty of evidence is low.^16^ We conducted a current state analysis and identified (1) an opportunity to improve interdisciplinary awareness of patients at high risk for pressure injury and (2) a lack of knowledge regarding the treatment plan for pressure injury prevention as primary drivers of pressure injuries. Although there is some literature on the benefit of interprofessional rounding in a PICU,^17^ there is a paucity of literature evaluating outcomes after implementation across all intensive care units (ICUs) within an institution. We aimed to implement weekly interdisciplinary ICU pressure injury rounds to increase awareness and planning for patients at high risk for pressure injury and reduce sHAPIs in all ICU patients. We aimed for a 10% reduction in sHAPIs across participating ICUs within 1 year of implementing interdisciplinary rounding.

## METHODS

This work was reviewed under institutional QI oversight by our hospital’s Quality and Safety Oversight Committee.

### Setting

This improvement work was conducted at an urban, quaternary, 364-bed (168 ICU beds) freestanding children’s hospital in downtown Chicago (IL). Our hospital is a member of the Children’s Hospital SPS Network, with access to SPS best practice bundle recommendations. At our hospital, pressure injury reduction work is overseen by the HAC Committee and is led by a hospital-wide pressure injury reduction team. This oversight committee includes nurses, physicians, certified wound ostomy nurses (CWONs), QI experts, respiratory care practitioners, and nutritionists, and has representation from all inpatient units across the hospital. We hypothesized that structured interdisciplinary rounds (IDRs) would improve multidisciplinary communication and shared awareness of patient-specific pressure injury risk, enabling earlier preventive interventions and reducing sHAPI.

### Context

Our institution had an established hospital-wide pressure injury prevention infrastructure through the HAC Committee. However, prevention activities within the ICUs were largely unit-based and reactive, creating an opportunity for a standardized interdisciplinary process focused on proactive identification of high-risk patients.

To more comprehensively identify opportunities for improvement in HAPI outcomes, a barriers assessment was performed. Subsequently, a key driver diagram was created (Fig. 1). The barriers assessment and key driver diagram involved interdisciplinary input, including bedside nurses, physicians, respiratory care practitioners, and occupational and physical therapists. The key driver diagram revealed multiple secondary drivers with opportunities to intervene via interdisciplinary rounding. Examples of such secondary drivers included increased shared awareness, CWON collaboration, and processes for the escalation of concerns.

**Fig. 1.**
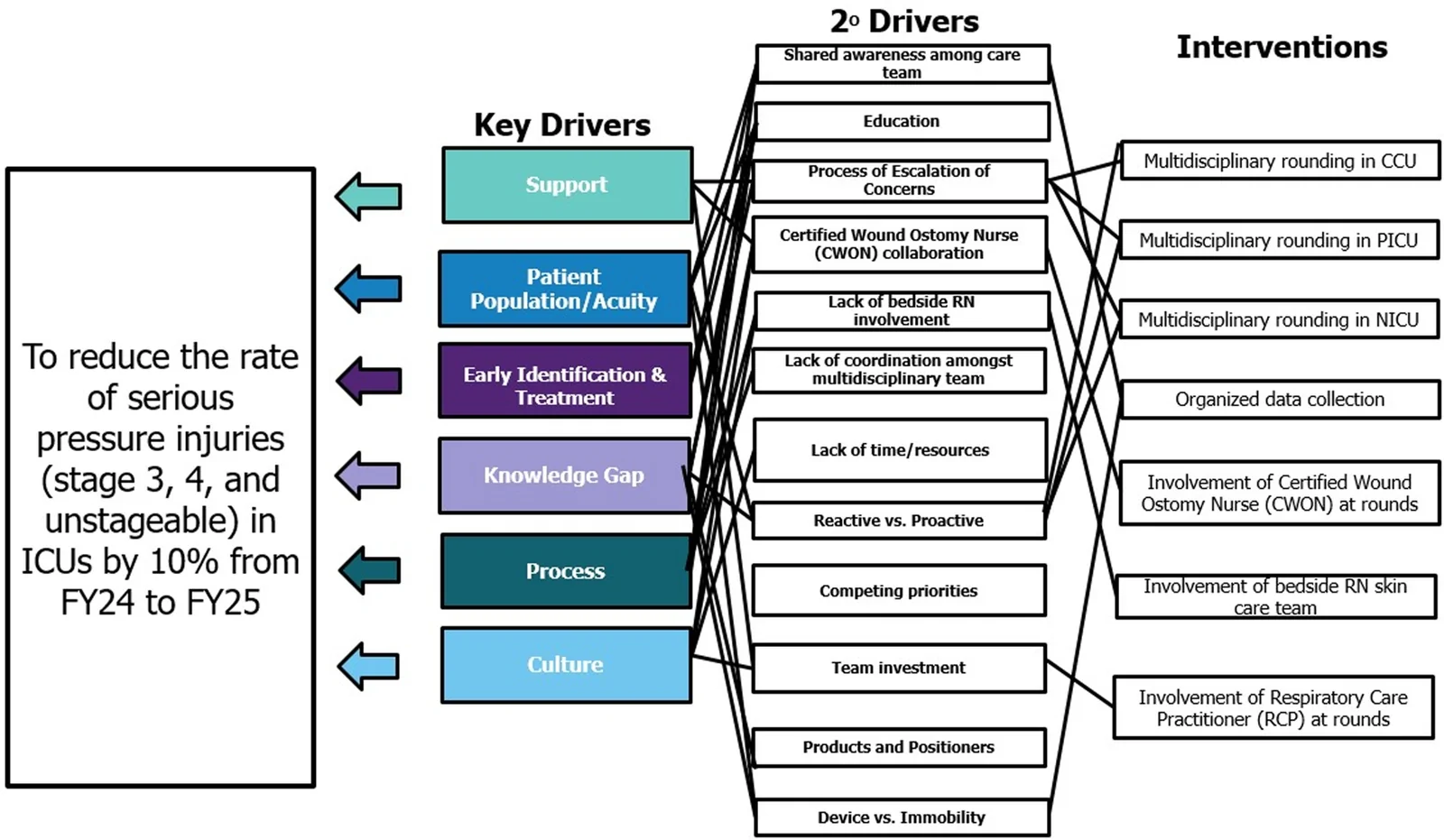
Key driver diagram showing multidisciplinary rounding to increase support, fill a knowledge gap, and support a change in culture over time. CCU, cardiac care unit; RN, registered nurse.

### Intervention: Interdisciplinary Rounding

This intervention was based on the premise that pressure injury prevention requires coordinated multidisciplinary care and that structured interdisciplinary communication improves the reliability and timeliness of preventive practices. Interdisciplinary pressure injury rounds were implemented in phases, beginning with weekly rounds in the CICU in October 2023 as a pilot. Following refinement through successive Plan-Do-Study-Act (PDSA) cycles, the intervention expanded to the NICU in October 2024 and the PICU in November 2024, resulting in different durations of intervention exposure across ICUs during the study period.

Each ICU HAPI IDR team comprises at least 1 attending physician, 1 nurse, a member of our CWON team, a clinical quality coordinator nurse, respiratory care practitioners, and a physical or occupational therapist. The respective ICU HAPI IDR teams meet weekly to discuss high-risk patients on each unit with the goal of implementing preventive interventions. Risk is determined based on the Braden QD Scale, a reliable and standardized tool to assess HAPI risk in pediatric patients, considering factors such as immobility and device placement.^18^ However, beyond the Braden QD, risk was also identified using clinical factors such as procedures (ie, continuous renal replacement therapy or ECMO), high-risk devices (ie, continuous bi-level positive airway pressure or continuous positive airway pressure), fluid overload, and mobility restrictions. Because IDR team members have clinical insight into the patients on the unit, they can identify at-risk patients that might otherwise not be identified by the Braden QD, for example, patients who are fluid overloaded or who experience hemodynamic instability with turning. This insight improves the effectiveness of risk assessment.

After each meeting, follow-up with the patient’s bedside team was prioritized to create awareness of a patient’s high-risk state and to implement the prophylactic measures that were identified as an opportunity during the rounding process. Each ICU accomplished this based on its individual communication practice and workflow.

### Proactive Patient Interventions

Following the ICU IDR team discussion of high-risk patients, a thorough proactive approach was adopted to prevent current pressure injuries from advancing or to prevent pressure injuries from occurring at all. Interventions were categorized into several themes. Interdisciplinary collaboration remained a prominent intervention. This included identifying specific patient needs and increasing awareness among primary team members and other involved consultants. Sedation and analgesia, the ongoing need for devices, and nutrition optimization were frequently discussed. Additionally, increased collaboration with the rehabilitation team and optimization of physical and occupational therapy orders and assessments were prominent themes. This allowed for appropriate specialty assessment and earlier mobility. Enhanced collaboration with respiratory care practitioners improved optimal device sizing and padding. At the bedside, multiple interventions were implemented, including surface optimization, prophylactic dressing application, increased patient assessments when needed, and specialized device assessments. Examples of these interventions include increased assessments for patients on continuous video electroencephalogram monitoring, application of sacral borders for immobile patients, and more frequent tracheostomy tie changes to relieve pressure and avoid a moist environment from secretions in patients with tracheostomies in place. Documentation optimization and direct one-on-one bedside education with the entire care team, from nursing assistants, bedside nurses, and frontline providers, were also frequent interventions.

### Iterative Improvement

There were 4 notable PDSA cycles associated with this effort. First, IDRs were expanded from the CICU to all other ICUs after a significant reduction in HAPIs in the CICU. The CICU first started HAPI rounds in October 2023. This was followed by the NICU in October 2024 and later the PICU in November 2024. During the second PDSA cycle, multiple iterations of practice occurred within each ICU to work toward an ideal functioning and data tracking state. These optimizations included the cadence of meetings, data storage, and editing the worksheet format of encrypted patient data. In the third PDSA cycle, we quickly recognized the value of having consistent CWON involvement on our teams. A CWON was assigned to a particular unit based on their experience. They joined, ensuring proactive consultation and follow-up on recommendations. During our fourth PDSA cycle, we increased involvement from other essential practitioners such as our skin care team and respiratory care practitioners on weekly rounds. Our skin care team consists of specialized nurses who round weekly, separate from IDRs, to confirm adherence to protocols, answer bedside nurse questions, and address new or high-risk wounds. Similarly, respiratory care practitioners were included, given a notable increase in device-related events from masks and prongs.

### Measurements

HAPI was defined in accordance with the SPS operational definition, guided by the National Pressure Injury Advisory Panel, and pressure injuries were staged accordingly. Serious pressure injuries (sHAPIs) were defined as stage 3, stage 4, and unstageable.

Outcome, process, and balancing metrics were identified at project onset. The primary outcome was the rate of sHAPIs across all ICUs. Secondary outcomes, including time between events and percentage of stage 1 and stage 2 HAPIs, were also assessed. Process metrics included the number of patients rounded on and the number of interventions implemented on a weekly basis. Staff rounding time burden and the number of non-sHAPI HACs were used as balancing metrics, as it was important that other HACs did not increase as a result of this increased focus on sHAPI prevention.

### Analysis

Because sHAPIs are relatively rare events, data were analyzed using a t-chart for each ICU to evaluate the time between sHAPIs. sHAPIs were tracked between September 2022 and November 2025 across all 3 units. Because each unit implemented rounding on a different date, baseline and intervention periods differed between each unit. Statistical process control chart rules were applied to each of the data sets, including 8 or more points above or below the centerline, indicating a centerline shift, and a single point outside the upper control limit or lower control limit, indicating special-cause variation.

For process metrics, a run chart was used to evaluate the number of patients rounded on and interventions implemented. The balancing metric on non-sHAPI ICU HACs was measured via a statistical process control c-chart.

## RESULTS

### Primary Outcome Metrics

The primary outcome, ICU sHAPI rate per 1,000 patient-days, decreased from 0.564 in fiscal year (FY)2024 (September 2023–August 2024) to 0.343 in FY2025 (September 2024–August 2025), representing a 39% reduction. The total number of ICU sHAPI events decreased from 24 to 15, whereas ICU patient-days increased from 42,530 to 43,737 during the same period.

### Secondary Outcome Metrics

Following implementation of IDRs, the CICU and PICU demonstrated 2 observations above the upper control limit, indicating special-cause variation, with longer intervals between sHAPI events and less frequent injuries (Figs. 2, 3). The NICU did not demonstrate special-cause variation during the study period, likely reflecting the low baseline frequency of sHAPIs and limited follow-up (Fig. 4). Among HAPIs occurring across the participating CICU, NICU, and PICU, the proportion classified as stage 1 and stage 2 increased from 60% in FY2024 to 68% in FY2025.

**Fig. 2.**
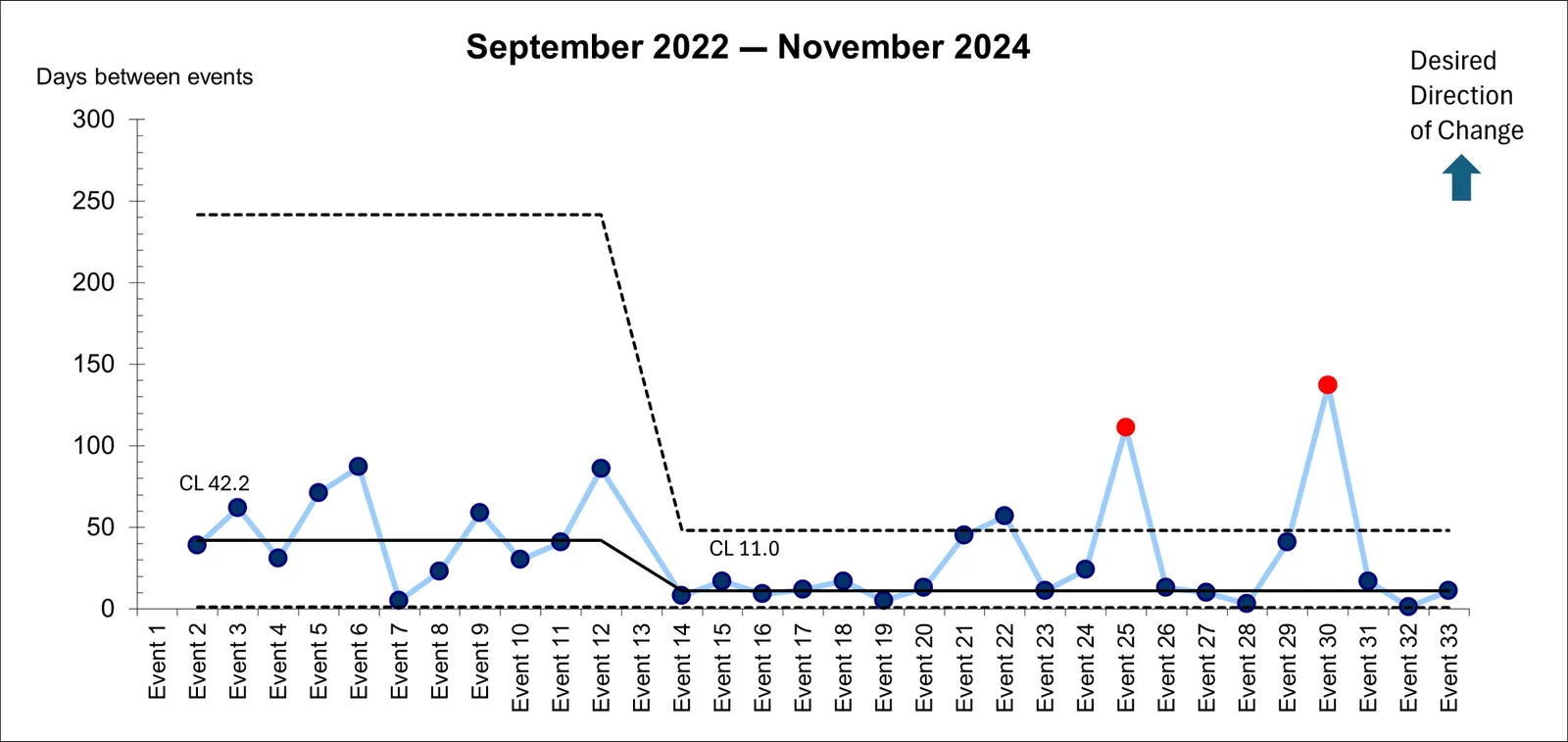
Days between sHAPIs in the PICU pre- and postintervention (T-chart). CL, control limit.

**Fig. 3.**
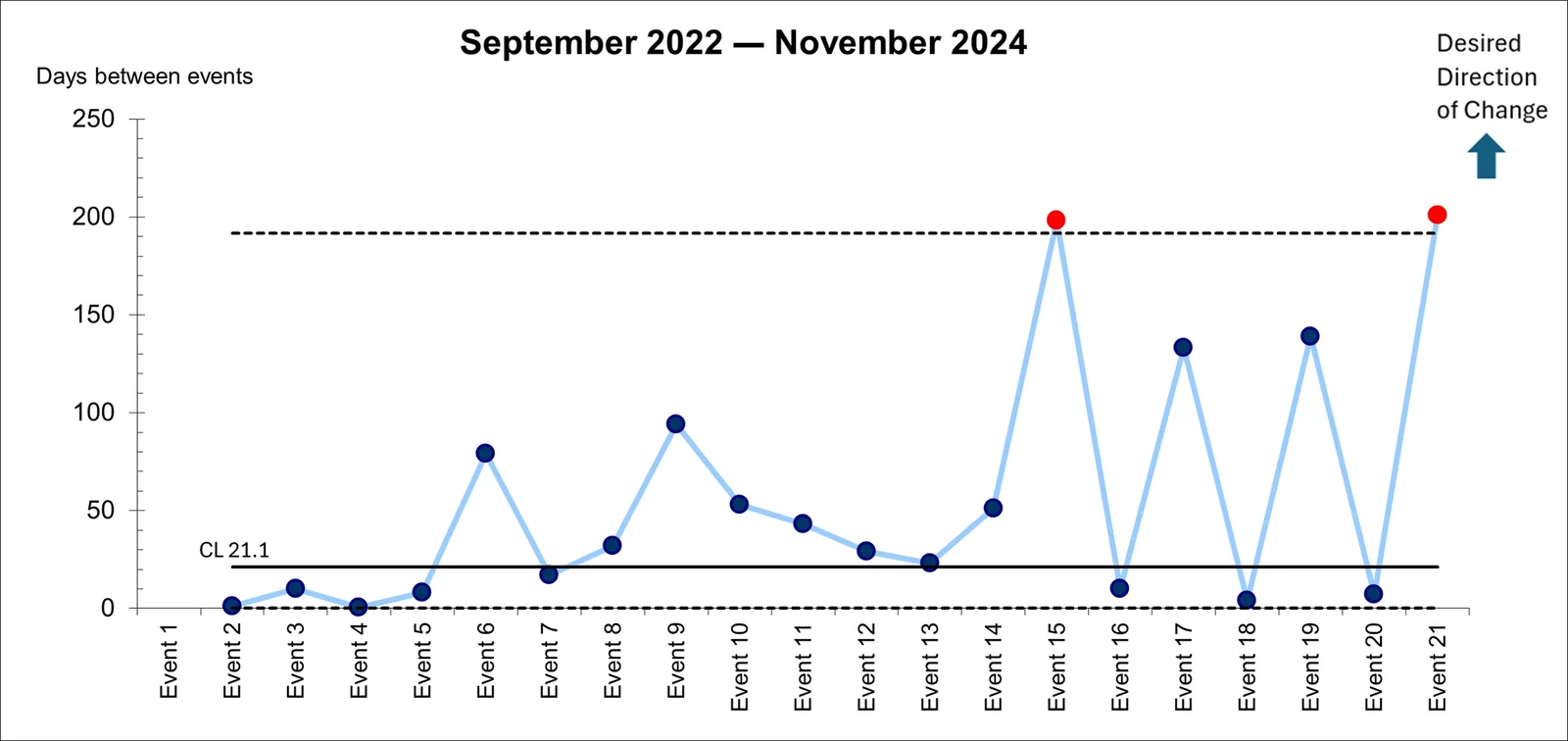
Days between sHAPIs in the CICU pre- and postintervention (T-chart). CL, control limit.

**Fig. 4.**
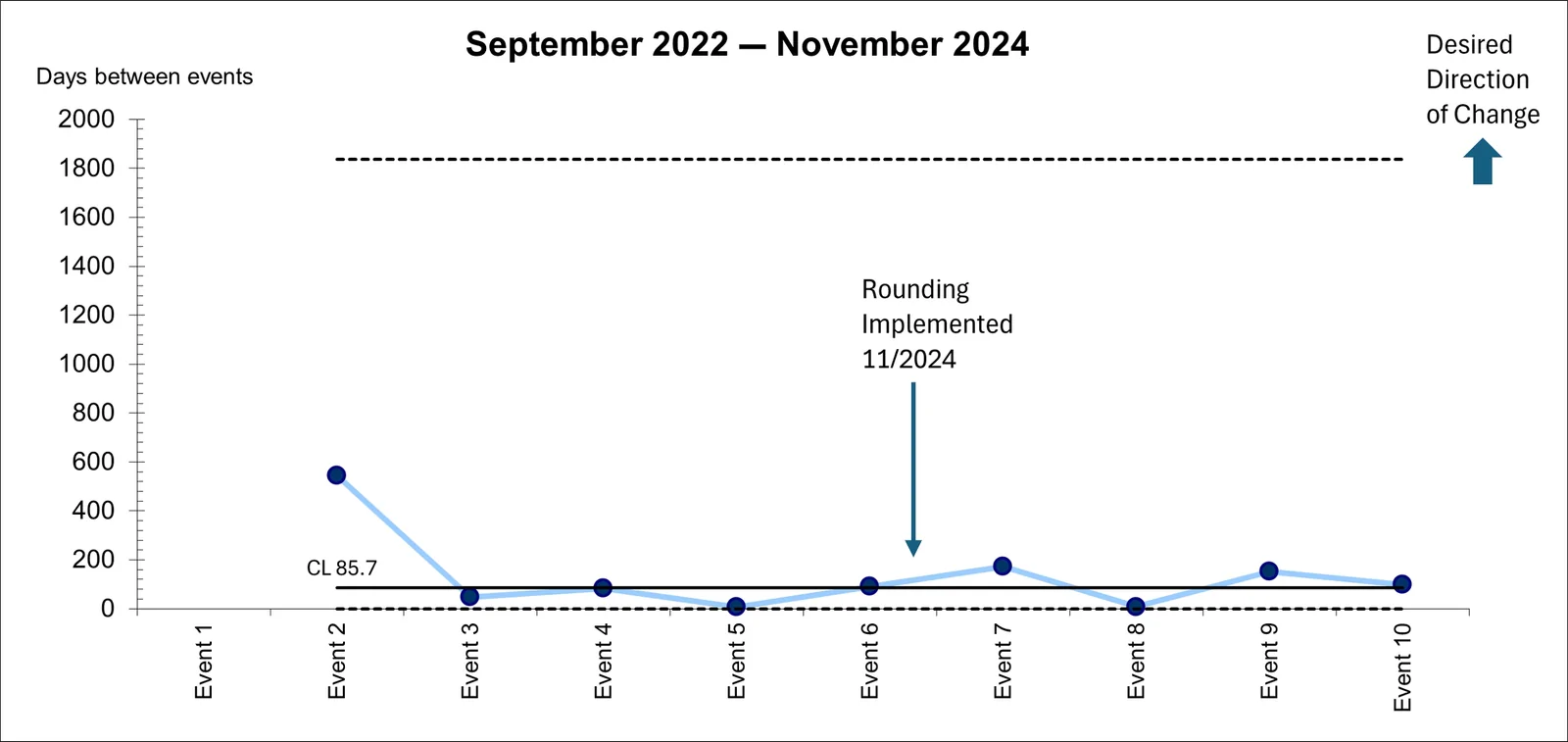
Days between sHAPIs in the NICU pre- and postintervention (T-chart). CL, control limit.

### Process Metrics

Process measures demonstrated increasing adoption of IDRs throughout implementation in the NICU and PICU (Fig. 5). As additional ICUs initiated the intervention, the number of patients discussed each week increased, accompanied by a corresponding increase in documented preventive interventions. More than 1,400 proactive interventions were implemented during the first year across the NICU and PICU.

**Fig. 5.**
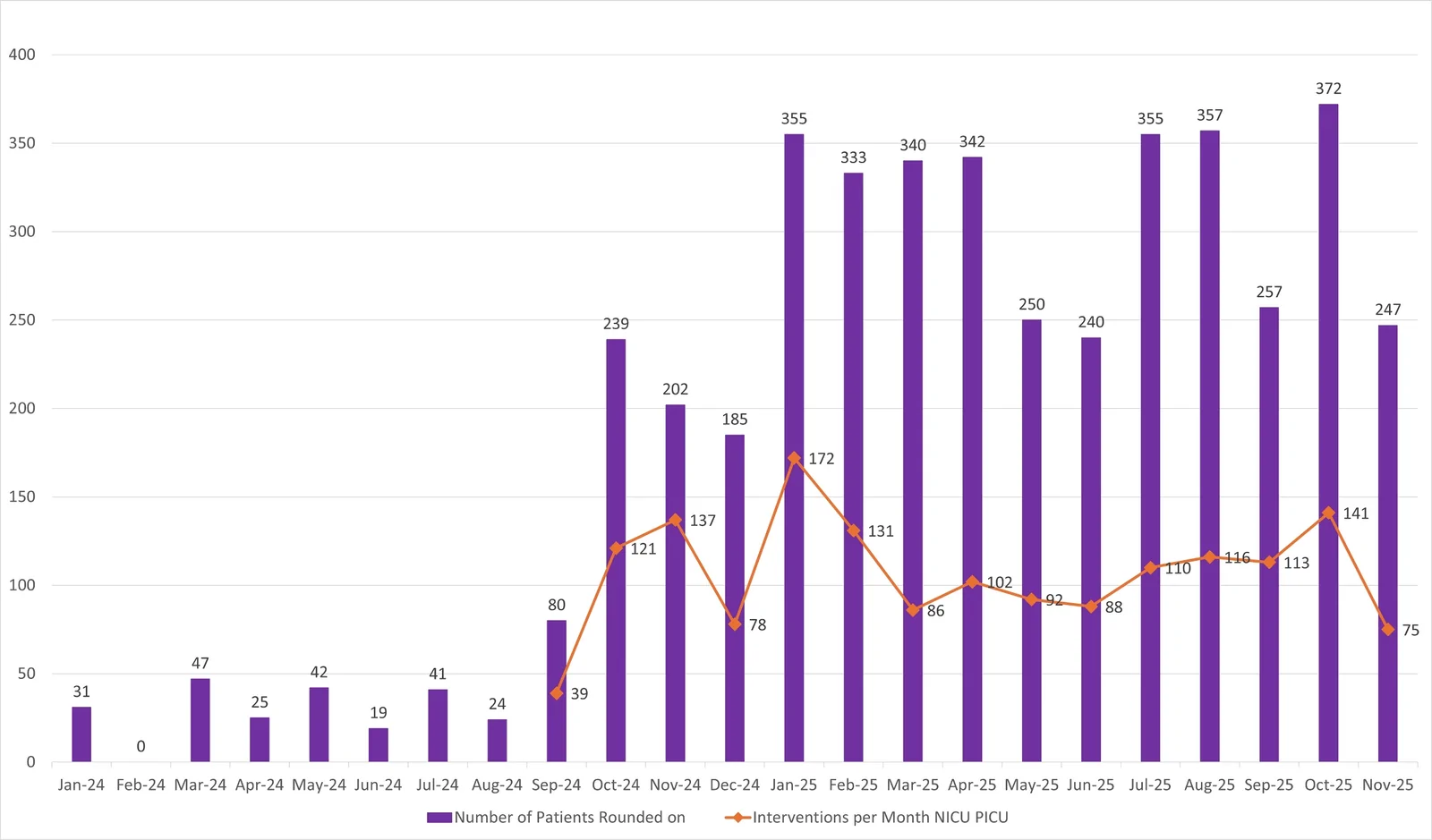
Number of patients rounded on and interventions implemented in the NICU and PICU each month.

### Balancing Metrics

Non-HAPI HACs (ie, central line associated blood stream infection, catheter associated urinary tract infection, and so on) did not increase during the first year of implementation; in fact, the number of events decreased. This decrease was significant, with a centerline shift from 4.8 to 2.8 events per month (Fig. 6). Specifically, as it relates to mobility, the unplanned extubation rate did not have a centerline shift and remained at 0.272 (October 2023–August 2025). Rounding time burden is important to consider, as each team rounded for approximately 1 hour once a week, totaling 184 hours of time. Additionally, participants in these rounds were not directly involved in patient care during HAPI rounds, ensuring that no clinical duties were missed.

**Fig. 6.**
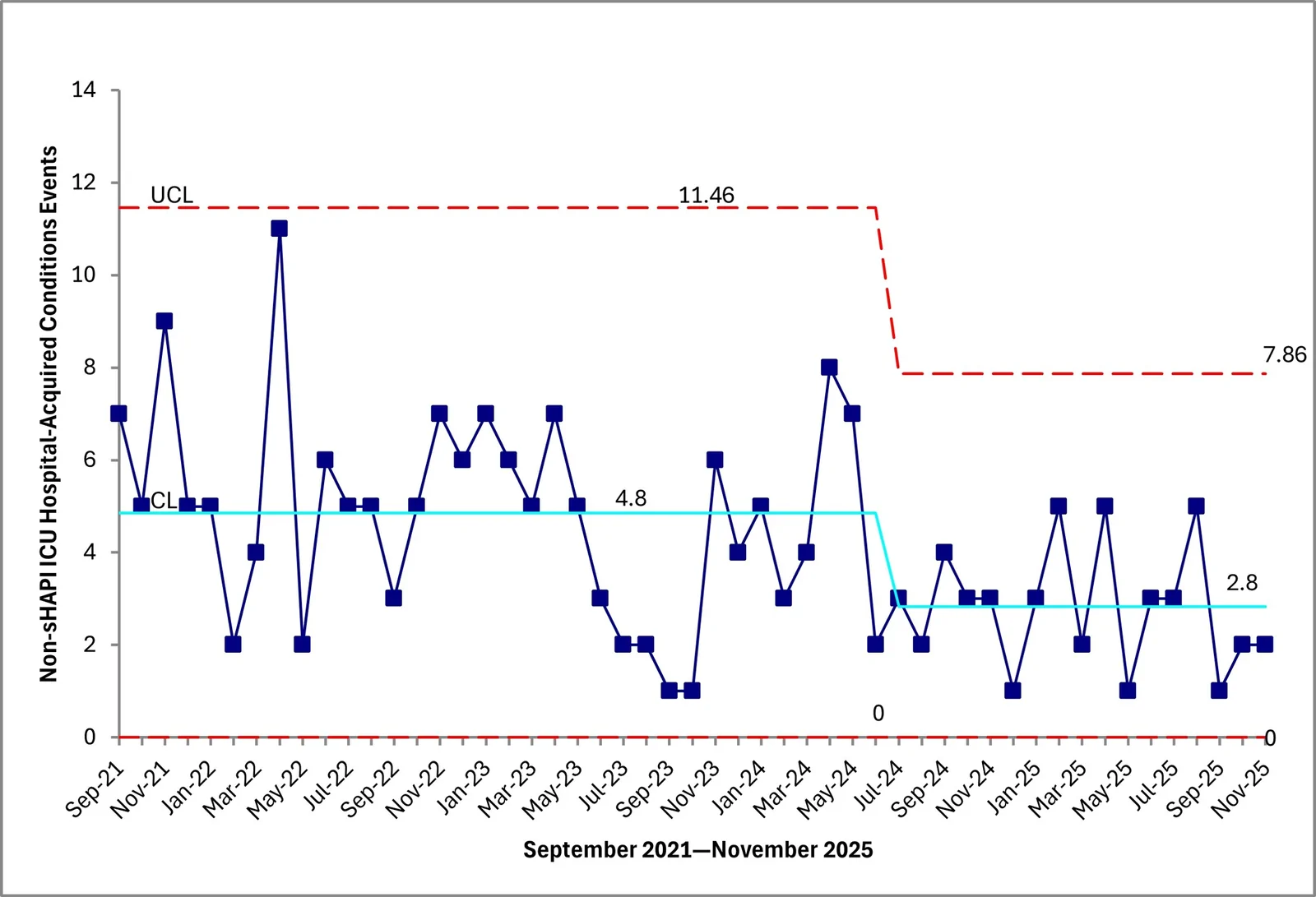
Non-sHAPI HACs pre- and postintervention. These include unplanned extubations, central line–associated bloodstream infections, catheter-associated urinary tract infections, peripheral intravenous extravasations, and falls. A centerline shift indicating a reduced rate of non-sHAPIs postintervention. CL, control limit.

## DISCUSSION

The aim of this QI study was to achieve a 10% reduction in the rate of sHAPIs per 1,000 patient-days across participating ICUs within 1 year of implementation. The adoption of interdisciplinary rounding directed at patient screening, discussion with care teams, implementing proactive interventions, and bedside education was associated with a reduction in harm beyond that expected using the SPS best practice bundle. Specifically, risk evaluation based on patient fluid status, device use, procedure requirement, and immobility was performed, expanding the assessment beyond the Braden QD. With this approach, a 39% reduction in sHAPI cases was observed. When performing a χ^2^ analysis of our primary outcome, the rate of ICU sHAPIs, no statistical significance was found. However, we achieved our institutional goal with a greater than 10% reduction in sHAPIs.

Because implementation occurred sequentially, the FY2025 institutional results reflect differing durations of intervention exposure across the 3 ICUs, with the CICU contributing the longest postimplementation period and the NICU and PICU contributing shorter follow-up periods. The proportion of lower stage (stages 1–2) HAPIs increased during this process; this finding may suggest earlier identification and proactive intervention to help prevent wound progression, although causation cannot be established. Additionally, the number of patients included and discussed in IDRs in both the NICU and PICU increased throughout this process (Fig. 5). The CICU did not track the weekly number of patients rounded on during this intervention period.

Through PDSA cycles, this rounding practice was expanded from the CICU to the PICU and NICU. Then, unit-specific data tracking systems were developed to ensure adequate capture of sHAPIs and subsequent interventions. Additionally, our interdisciplinary team was expanded to include other key members to help support sHAPI prevention. Integration of CWONs, skin care team, and respiratory care practitioners into our weekly rounds highlighted the importance of having shared awareness and buy-in from multiple practitioners to achieve a thoughtful approach to patient care. Given that sHAPIs continue to be a rare event in the ICUs, data from all 3 units were analyzed using a t-chart. The CICU and PICU both showed longer time intervals between sHAPIs events, indicating that events were becoming rarer following our intervention. This was not appreciated in the NICU. We believe this is because sHAPIs were already rarer events at baseline. It will likely require more time to show the impact of our interventions in the NICU.

Because the intervention was introduced sequentially across ICUs, the aggregate fiscal-year comparison should be interpreted as the cumulative institutional experience during phased implementation rather than equivalent postintervention follow-up across all units. FY2024 included substantial CICU intervention exposure; however, this fiscal-year comparison should not be interpreted as a traditional pre-/postintervention analysis. The accompanying unit-level statistical process control charts provide additional context by evaluating changes relative to each unit’s implementation timeline.

It is important to note that as sHAPI rates decreased, other non-sHAPI HAC rates had no observed increase. Non-sHAPI events demonstrated special-cause improvement during the study period, with 7 consecutive monthly observations below the original centerline from July 2024 through January 2025, prompting a centerline shift from 4.8 to 2.8 (Fig. 6). This temporal pattern occurred following implementation of our proactive sHAPI interventions and coincided with a broader institutional focus on proactive prevention of all HAC (central line associated blood stream infection, catheter associated urinary tract infection, unplanned extubation).

To promote sustainability, weekly interdisciplinary pressure injury rounds have been incorporated into routine ICU operations with defined representation from nursing, physicians, CWONs, rehabilitation, respiratory care, and quality personnel. Standardized rounding processes, unit-specific workflows, and ongoing performance review through the HAC Committee support continued implementation and facilitate spread of best practices across ICUs.

## LIMITATIONS

This project has several limitations. First, standardizing data collection across multiple ICUs was challenging, likely resulting in underestimation of both the number of patients discussed and preventive interventions implemented. It is possible that those patients who were admitted to an ICU for less than 7 days were not rounded on. However, all patients who developed pressure injuries had been identified as high risk and rounded on before the development of their HAPI. Long-term follow-up of interventions was also limited. Inconsistent nursing participation due to clinical staffing needs occasionally hindered communication and reinforcement of recommended interventions, highlighting opportunities to improve handoffs and maintain a shared understanding of patient-specific risk.

Second, 2 hospital-wide initiatives may have also influenced pressure injury outcomes. A high-risk HAC consult process was introduced in the NICU in June 2024 and expanded hospital-wide in the spring of 2025, whereas new specialty beds were introduced in May 2025. Because these initiatives overlapped with IDRs, their independent effects cannot be separated. However, the staggered implementation of IDRs and unit-level statistical process control analyses provide temporal context for the observed improvements. Future studies should evaluate the independent and additive effects of these interventions. Finally, the relatively short study period and infrequent occurrence of sHAPIs limited our ability to detect sustained statistical improvement, particularly in units with low baseline event rates.

## CONCLUSIONS

Interdisciplinary rounding is an established strategy that promotes collaboration among healthcare professionals and has been associated with improved patient outcomes and reductions in HACs.^19–21^ In this initiative, standardized interdisciplinary skin rounds were successfully integrated into routine workflow across multiple ICUs. Combined with screening, this approach shifted pressure injury prevention from reactive treatment to proactive risk identification and was associated with fewer sHAPIs, supporting interdisciplinary rounding as a practical and scalable prevention strategy.
